# Nonpharmacologic Interventions in Prevention and Treatment of Hypertension

**DOI:** 10.1155/2019/6709817

**Published:** 2019-03-13

**Authors:** Omotayo O. Erejuwa, Siew Hua Gan, Andrea M. P. Romani, Mohammad A. Kamal, Srinivas Nammi

**Affiliations:** ^1^Department of Pharmacology & Therapeutics, Faculty of Clinical Medicine, Ebonyi State University, Ebonyi State, Nigeria; ^2^School of Pharmacy, Monash University Malaysia, Jalan Lagoon Selatan, 47500 Bandar Sunway, Selangor, Malaysia; ^3^Case Western Reserve University, Cleveland, USA; ^4^King Fahd Medical Research Center, King Abdulaziz University, P.O. Box 80216, Jeddah 21589, Saudi Arabia; ^5^Enzymoics, 7 Peterlee Place, Hebersham, NSW 2770, Australia; ^6^Novel Global Community Educational Foundation, Australia; ^7^School of Science and Health, USA; ^8^NICM Health Research Institute, Western Sydney University, NSW 2751, Australia

Hypertension contributes to high rates of mortality and morbidity in both developing and developed countries. It predisposes individuals to several chronic diseases including stroke and other cardiovascular diseases. Predisposing factors such as sedentary lifestyles, unhealthy dietary habits, and physical inactivity are associated with its growing prevalence over the years. Besides social-economic factors, increased incidence of diabetes and obesity as well as modernized lifestyles plays important roles in the growing prevalence of uncontrolled hypertension, in particular in developing countries.

Currently, despite availability of several antihypertensive drugs, attaining an adequate blood pressure control is still a challenge. This can be attributed to several factors. For example, many of the existing antihypertensive agents do not modulate oxidative stress, which is an important pathophysiologic pathway for the development of hypertension and several of its complications. Poor medication adherence, reduced drug effectiveness, drug unaffordability or unavailability, and side/adverse effects of medications are additional important obstacles that weigh significantly in our ability to control the disease. To address some of these limitations, attention has been paid in recent years to the use of various nonpharmacologic interventions as a more viable alternative to treat the disease.

This special issue reports on the current status of nonpharmacologic approaches taken thus far across the world, in the prevention and treatment of hypertension. Several contributions were received from researchers in most of the continents. After stringent and thorough reviewing process, nine manuscripts (six original research, one clinical study, and two review articles) were accepted for publication.

H. H. Alhawari et al. assessed the frequency of hypertension among healthy university students and its association with gender, body mass index (BMI), smoking, and family history of both hypertension and cardiovascular diseases. They found significant gender differences in both systolic and diastolic blood pressure (i.e., higher in males as compared to females). Upon comparison of the mean difference in both systolic and diastolic blood pressure with BMI, there were significant differences in both systolic and diastolic blood pressure. First-degree family history of both hypertension and cardiovascular diseases was also found to affect systolic but not diastolic blood pressure. G. Musinguzi et al. investigated and identified factors influencing compliance and health seeking behavior among hypertensive patients, being related to health systems and patient socioeconomic and structural environment. These include self-medication and self-prescription of antihypertensive drugs as well as marketing of herbal remedies. H. Javadzade et al. assessed the effectiveness of a theory-based self-care intervention with the application of health literacy approaches in hypertensive patients and limited health literacy. The results have a potential to assist with the development of a theoretical framework for self-care intervention in patients with high blood pressure and limited health literacy. T. M. Dokunmu et al. assessed the risk of developing hypertension in healthy adult Nigerian population. Prehypertension was found in 36.8% population and elevated blood pressure in 31% individuals with hypertensive symptoms. The researchers found that age more than 35 years was an independent risk and this increases to 26.48 in the presence of prediabetes and random blood glucose greater than 100 mg/dL. Large scale screening and management of hypertension are recommended to reduce the burden of the disease. A. Larki et al. determined the factors that influence adherence to self-care behaviors among low health literacy hypertensive patients based on health belief model. The researchers found that designing and implementing educational programme to increase self-efficacy of patients and promote their beliefs about perceived susceptibility and severity of complications may improve self-care behaviors among low health literacy hypertensive patients.

E. Drevenhorn proposed a middle-range theory of nursing in hypertension care. Patients related concepts such as attitude and beliefs concerning health were presented. The researcher highlighted the clinical and research implications of the theory. Hypertension is a chronic disease that necessitates long-term self-management. Using a semistructured interview guide, K. D. Wright et al. described the process in which African American older adults and nurse researchers cocreated an intervention to address stress in the self-management of hypertension. Based on participants feedback, four biweekly (2-hour) group sessions which incorporated patients suggestions and concerns were created. Though it may be of significant benefits, further studies are required to test the generalizability of this technique. M. Horiuchi et al. investigated the effects of resistance exercise under hypoxia on postexercise in eight healthy young males. The findings suggested that hypoxic condition elicits greater impact on hypotension following resistance exercise (in comparison to normoxia). The data also suggest that the underlying mechanisms for the attenuation of hypotension after resistance exercise may vary between normoxia and hypoxia. Romani presented a review paper that underscores the beneficial effects of magnesium on blood pressure maintenance.

The issue highlights the importance of treatment and control of blood pressure via nonpharmacologic approaches. It thus reinforces the need for researchers to intensify more research in this aspect of hypertension management. This may help to reduce drastically the increasing prevalence and burden of hypertension worldwide.

