# Erratum to “Nonpharmacologic Interventions in Prevention and Treatment of Hypertension”

**DOI:** 10.1155/2019/2907675

**Published:** 2019-10-13

**Authors:** Omotayo O. Erejuwa, Siew Hua Gan, Andrea M. P. Romani, Mohammad A. Kamal, Srinivas Nammi

**Affiliations:** ^1^Department of Pharmacology & Therapeutics, Faculty of Clinical Medicine, Ebonyi State University, Ebonyi State, Nigeria; ^2^School of Pharmacy, Monash University Malaysia, Jalan Lagoon Selatan, 47500 Bandar Sunway, Selangor, Malaysia; ^3^Case Western Reserve University, Cleveland, USA; ^4^King Fahd Medical Research Center, King Abdulaziz University, P.O. Box 80216, Jeddah 21589, Saudi Arabia; ^5^Enzymoics, 7 Peterlee Place, Hebersham, NSW 2770, Australia; ^6^Novel Global Community Educational Foundation, Australia; ^7^School of Science and Health, Western Sydney University, NSW 2751, Australia; ^8^NICM Health Research Institute, Western Sydney University, NSW 2751, Australia

In the article titled “Nonpharmacologic Interventions in Prevention and Treatment of Hypertension” [1], there was an error in the affiliation of the fifth author. The corrected authors' list is shown above.

## References

[1] Erejuwa O. O., Gan S. H., Romani A. M., Kamal M. A., Nammi S. (2019). Nonpharmacologic interventions in prevention and treatment of hypertension. *International Journal of Hypertension*.

